# Supplementary material for: HIVprotI: an integrated web based platform for prediction and design of HIV proteins inhibitors
Source: J Cheminform. 2018 Mar 9;10:12. doi: 10.1186/s13321-018-0266-y (PMC5845081; doi:10.1186/s13321-018-0266-y)
Supplement: Supplementary file 2 — Additional file 2. Source code of HIVProtI web server. [file 13321_2018_266_MOESM2_ESM.zip › HIVprotI_Source-code/hivprotI/weka-3-6-6/doc/weka/attributeSelection/UnsupervisedAttributeEvaluator.html]

UnsupervisedAttributeEvaluator


---


|  |  |  |
| --- | --- | --- |
| **Overview** \| **Package** \| **Class** \| **Tree** \| **Deprecated** \| **Index** \| **Help** \| **Weka's home** | |  |
| **PREV CLASS**   **NEXT CLASS** | **FRAMES**    **NO FRAMES**     **All Classes** |  |
| SUMMARY: NESTED \| FIELD \| CONSTR \| METHOD | DETAIL: FIELD \| CONSTR \| METHOD |  |


---


## weka.attributeSelection Class UnsupervisedAttributeEvaluator

```
java.lang.Object
  weka.attributeSelection.ASEvaluation
      weka.attributeSelection.UnsupervisedAttributeEvaluator
```

**All Implemented Interfaces:**: java.io.Serializable, AttributeEvaluator, CapabilitiesHandler, RevisionHandler

**Direct Known Subclasses:**: LatentSemanticAnalysis, PrincipalComponents

---

``` public abstract class UnsupervisedAttributeEvaluator extends ASEvaluation implements AttributeEvaluator ```

Abstract unsupervised attribute evaluator.

**Version:**
:   $Revision: 1.10 $

**Author:**
:   Mark Hall (mhall@cs.waikato.ac.nz)

**See Also:**: Serialized Form

---

**Constructor Summary**

`UnsupervisedAttributeEvaluator()`


**Method Summary**

**Methods inherited from class weka.attributeSelection.ASEvaluation**

`buildEvaluator, forName, getCapabilities, getRevision, makeCopies, postProcess`

**Methods inherited from class java.lang.Object**

`equals, getClass, hashCode, notify, notifyAll, toString, wait, wait, wait`

**Methods inherited from interface weka.attributeSelection.AttributeEvaluator**

`evaluateAttribute`

**Constructor Detail**

### UnsupervisedAttributeEvaluator

```
public UnsupervisedAttributeEvaluator()
```


---


|  |  |  |
| --- | --- | --- |
| **Overview** \| **Package** \| **Class** \| **Tree** \| **Deprecated** \| **Index** \| **Help** \| **Weka's home** | |  |
| **PREV CLASS**   **NEXT CLASS** | **FRAMES**    **NO FRAMES**     **All Classes** |  |
| SUMMARY: NESTED \| FIELD \| CONSTR \| METHOD | DETAIL: FIELD \| CONSTR \| METHOD |  |


---
